# Perceptions of facilitators, barriers and adaptations for provision of obstetric fistula surgery and care: a qualitative descriptive study of service providers in selected hospitals in Busoga sub-region, eastern Uganda

**DOI:** 10.3389/fgwh.2026.1813512

**Published:** 2026-06-03

**Authors:** Peter Kivunike Mukasa, Mary Kinney, Wilberforce Mugwanya, Andrew Kivunike, Abdmagidu Menya, Vallence Ngabo Maniragaba, Debra Jackson

**Affiliations:** 1 School of Public Health, University of the Western Cape, Bellville, South Africa; 2 Global Surgery Division, Department of Surgery, University of Cape Town, Cape Town, South Africa; 3 United Nations Population Fund (UNFPA), Uganda Country Office, Kampala, Uganda; 4 School of Social Sciences, College of Humanities and Social Sciences, Makerere University, Kampala, Uganda; 5 Medical Research Council/ Uganda Virus Research Institute and London School of Hygiene and Tropical Medicine Uganda Research Unit, Entebbe, Uganda; 6 Mbarara University of Science and Technology, Mbarara, Uganda; 7 London School of Hygiene and Tropical Medicine, London, United Kingdom

**Keywords:** adaptations, barriers, Busoga sub-region, facilitators, female genital fistula, fistula repair, fistula treatment, obstetric fistula

## Abstract

**Introduction:**

Obstetric fistula is a devastating childbirth injury common in low-resource settings, with many women failing to access surgical repair due to health system challenges. However, limited evidence exists on the health system factors shaping the provision of fistula care and surgery. The study aimed to explore facilitators, barriers encountered, and adaptation mechanisms employed by healthcare providers in the provision of fistula surgery and care.

**Methods:**

We conducted 20 qualitative interviews in two hospitals providing fistula surgery and care in the Busoga sub-region, a government and a private not for profit hospital between June and December 2024. In-Depth Interviews (*n* = 12) were conducted with purposively selected healthcare providers and facility administrators. We also conducted Key Informant Interviews (*n* = 8) with purposively selected national fistula experts/surgeons, policy makers and implementing partners. Data were audio-recorded, transcribed verbatim and analyzed using a deductive thematic analysis, guided by the World Health Organization (WHO) health system blocks framework.

**Results:**

Seven themes emerged from the findings including: 1) human resources, 2) equipment and supplies, 3) infrastructure, 4) financial resources, 5) access to services, 6) adaptation mechanisms, and 7) recommendations for improved provision of obstetric surgery and care. Facilitators to provision of fistula surgery and care included government coordination and stewardship, availability of trained specialists during fistula camps, donor-supported equipment and supplies, community mobilization, and financial support to patients. However, significant barriers including inadequate and poorly compensated staffing, high staff turnover, inconsistent supplies and equipment, limited ward space leading to overcrowding, and an overreliance on external donor funding constrained routine service provision. Access barriers included stigma, limited awareness and high transport costs involved in seeking care. However, the hospitals have developed adaptation mechanisms such as flexible scheduling and improvising spaces for instance use of tents to cope with bed and space shortages. Participants recommended improving the provision of care through better preparation and staffing of hospitals.

**Conclusion:**

While fistula camps provide short-term access to life changing fistula surgery, they reflect underlying health system challenges. Strengthening routine fistula services by addressing systemic, financial, and sociocultural barriers is critical to ensure equitable and sustainable fistula care in Uganda.

## Introduction

Childbirth morbidity, including obstetric fistula, accounts for over 11% of the global surgical burden, creating a significant gap in unmet need for surgical services, especially in low and middle-income countries (LMICs), including Uganda (1, 2). Obstetric fistula is a tragic life-changing birth injury affecting many mothers in LMICs. It is caused by prolonged, obstructed labor without timely access to medical intervention, resulting in an abnormal opening between the birth canal and bladder and/or rectum. This condition leads to uncontrolled leakage of urine and/or feces (3–9). Globally, it is estimated that at least 500,000 women and girls, mostly in LMICs, are affected by obstetric fistula with approximately 30,000 to 130,000 new cases arising every year (10, 11).

In Uganda, obstetric fistula remains a significant public health issue, with over 75,000 women and girls currently living with the condition and about 1,900 new cases reported annually (12–18). Efforts are underway to prevent, treat, and reintegrate women with obstetric fistula and other birth injuries through the implementation of the Uganda National Obstetric Fistula Strategies. These initiatives focus on strengthening the prevention of obstetric fistula and enhancing the capacity for fistula repair and treatment within healthcare systems. Currently, fistula services are provided predominantly at national and regional referral hospitals, but also a few general district hospitals, health centers and selected private not-for-profit (PNFP) hospitals (13, 14).

Care and treatment for fistula is limited due to inadequate number of surgeons and resources leading to an unmet need for repair as high as 90% (19–21). Programs aimed at addressing this substantial obstetric fistula backlog face systemic gaps, compounded by a high volume of surgical emergencies in hospitals. Only about 19% of hospitals in Uganda (28 out of 146 hospitals) can provide fistula surgery services, mostly through repair camps, and very few of these hospitals are able to offer routine fistula repairs (15, 19, 20).

The National Obstetric Fistula Strategy underscores integration of routine fistula care and surgery into facility-based services, and if operationalized, could greatly impact reducing the huge fistula backlog. However, despite the availability of free healthcare services for obstetric fistula repair in the available treatment centers across the country, significant challenges and gaps remain in terms of the provision of fistula treatment and repair services (15, 22, 23). Consequently, many women and girls with fistula continue to suffer medically, socially and some even die without receiving treatment (15, 21, 24, 25).

Routine repairs are often not conducted in hospitals due to a lack of skilled human resources, supplies, and equipment, alongside overwhelming numbers of emergencies. Additional access barriers for clients include long distances to facilities, poverty, lack of information and stigma (20). Providing essential surgical services, especially for women living with fistula, is critical in restoring their hope and dignity (21, 24).

Most studies reviewed have focused on barriers to accessing obstetric treatment from the perspective of women living with obstetric fistula (26–29). Seeking expert knowledge and perspectives from service providers, hospital managers and policy makers is important to optimize and forge practical ways to operationalize routine fistula in targeted hospitals across the country. Health care providers who work in these district, regional and national referral hospitals are strategically placed to offer perspectives on the facilitators, barriers, adaptation mechanisms and practical recommendations to offer routine fistula repairs.

There is limited data on the service providers perceptions of barriers, facilitators and adaptations for rollout of routine obstetric fistula surgery and care in Uganda yet this is important in improving care for women with this debilitating condition. Therefore, this study was designed to explore the facilitators, barriers encountered and adaptation mechanisms enforced by healthcare providers in the provision of fistula surgery and care.

## Materials and methods

This section describes the research setting, the participants, data collection, data analysis and the obtaining of ethical approval. It should be noted that the data in this paper are reported in accordance with the Standards for Reporting Qualitative Research (SRQR) using the consolidated criteria for reporting qualitative studies (COREQ) (30).

### Study design

This study used a descriptive qualitative approach to explore the facilitators, barriers and adaptive mechanisms by healthcare providers in the provision of fistula surgery and care. In-Depth Interviews (*n* = 12) were conducted with healthcare providers, including fistula nurses and midwives, fistula surgeons and other specialists (surgeons, obstetricians, urologists), as well as hospital directors and administrators, between June and December 2024. We also conducted Key Informant Interviews (*n* = 8) with national fistula experts and surgeons, policy makers and implementing partners. The Key informants were selected based on guidance from the Fistula Technical Working Group (FTWG). This paper focuses on data from healthcare professionals involved in fistula surgery and care, administrators and policy makers.

### Study setting

The study was conducted in two hospitals located in the Busoga sub-region in eastern Uganda. Given the limited number of hospitals providing fistula care and the small number of healthcare workers involved in fistula care at the study sites, participants could easily be identifiable if specific hospitals were named. To maintain confidentiality, the hospitals are referred to as Hospital A, a government/public hospital, and Hospital B, a private not-for-profit (PNFP) facility. Both hospitals are key centers for obstetric fistula surgery and care within the sub-region.

The Busoga sub-region is one of the lowest performing in Uganda in terms of healthcare service delivery and among the regions with the highest prevalence and incidence of obstetric fistula in the country (18, 19). Uganda's health sector care system is structured in levels of care delivery at national, regional, district, and sub-district levels, with fistula care integrated in the system through defined interventions at each level, as shown in **(**Table 1**)**.

**Table 1:** Task distribution of Fistula care by level of healthCare delivery system.

Level	Tasks
HCII and III	Primary prevention, case detection, awareness creation, community mobilization, follow-up and referral for treatment
HC IV	Primary and secondary prevention, case detection, awareness creation and community mobilization, follow-up and referral for treatment
General Hospitals	Primary and secondary prevention, case detection, awareness creation and community mobilization, follow up, simple fistula repairs and referral of complex cases for treatment, carry out monitoring and evaluation
Regional Referral Hospitals	Primary and secondary prevention, case detection, awareness creation and community mobilization, follow-up and referral for treatment, repair complex fistula repairs, implement out-reach fistula care programs, training and technical supervision for fistula care programs in the region.
National Referral Hospitals	Primary and secondary prevention, case detection, awareness creation and community mobilization, follow up and referral for treatment, repair complex fistula repairs, implement out-reach fistula care programs, training and technical supervision for fistula care programs in the country.
CHEWs	Case detection, awareness creation, community follow up, mobilization and referral for treatment

Source. National Obstetric fistula strategy 2021–2026.

At both hospitals, fistula surgery and care services are mainly offered through periodic fistula repair campaigns or “fistula camps” organized by the Ministry of Health in collaboration with support from development partners. The details of the two facilities regarding fistula services are shown in Table 2.

**Table 2:** Shows the details of the two hospitals and the fistula services offered.

Hospital	Facility level	Catchment area	Fistula services provided
Hospital A	Government hospital	Serves an estimated population of 2,000,000 people	Primary and secondary prevention, case detection, awareness creation and community mobilization, follow-up and referral for treatment, repair complex fistula repairs, implement out-reach fistula care programs, training and technical supervision for fistula care programs in the region.
Hospital B	PNFP hospital	Serves an estimated population of 500,000 people	Primary and secondary prevention, case detection, awareness creation and community mobilization, follow-up and referral for treatment, repair complex fistula repairs, implement out-reach fistula care programs, training and technical supervision for fistula care programs in the country.

### Study population

The study was conducted among healthcare professionals and administrators involved in fistula surgery and care at both hospitals. Prior visits and calls were made to the sites and participants to explain the data collection exercise and agree convenient time and will be good for the interviews, cognizant of their busy schedules. Participants included fistula midwives, fistula nurses, fistula specialists (surgeons, obstetricians, urologists), as well as hospital directors and administrators. We also interviewed other national fistula surgeons/experts, civil society partners and policymakers from the Ministry of Health and United Nations Agencies as key informants.

### Recruitment

A total of 20 participants were interviewed for this study to explore their perceptions of facilitators, barriers and adaptations for the provision of obstetric fistula surgery and care. We conducted 12 In-Depth Interviews (IDIs) with healthcare providers and facility administrators: Hospital A (*n* = 6) and Hospital B (*n* = 6). The participants included fistula midwives (*n* = 2), fistula nurses (*n* = 4), specialist doctors, surgeon/obstetrician/urologist) (*n* = 2), hospital administrators (*n* = 2), and hospital directors (*n* = 2).

Hospital administrators are personnel involved in the day-to-day operational management at the hospitals, such as ward coordination and program implementation, while hospital directors were senior leaders responsible for strategic oversight and planning at the facility level. Participants were purposefully selected based on their direct involvement and unique roles in fistula care at clinical, programmatic and policy levels. With the help of the hospital's administration, the research team obtained a list of healthcare workers and administrators involved in fistula care. These purposively selected healthcare and administrators who support fistula services at both hospitals were interviewed.

To obtain insights beyond the two facilities, we also conducted eight (8) KIIs with fistula surgeons and other specialists (surgeons, obstetricians and urologists) (*n* = 4), implementers/donors/partners (*n* = 2), and policymakers from the Ministry of Health (*n* = 2). Key informants were purposefully selected based on guidance from the Fistula Technical Working Group of the Ministry of Health. None of the identified participants from either category declined to participate in the study.

### Data collection

Interviews were conducted face-to-face from June to December 2024 with healthcare providers, hospital managers, partners and policymakers involved in fistula care. Interviews with healthcare providers were conducted at the participants’ respective hospitals during fistula repair camps that were organized by the Ministry of Health. Key informants were interviewed during the fistula camps, while national-level key informants were interviewed at their respective workplaces.

A semi-structured interview guide (Supplementary material 1) was developed using the WHO building blocks that were used as the organizing framework and pilot-tested to ensure clarity, consistency and relevance to the study objectives. Interviews were conducted in the participants’ preferred language (English or Lusoga, a local dialect) by trained and experienced qualitative researchers (PKM, AM, and AKM), all with master's degrees, with PKM also being a doctoral student.

All participants provided written informed consent before their participation. All interviews took place in a private and quiet setting that was convenient for the participants to ensure confidentiality and lasted between 30 and 60 min. All interviews were audio-recorded and field notes were taken to complement the recordings. Participants who declined to be audio recorded gave consent for written notes to be taken instead.

### Data management and analysis

All interviews were transcribed verbatim. Interviews conducted in the local language, Lusoga (*n* = 5), were translated into English by experienced bilingual members of the research team (AM and AK), who are fluent in both languages. The translation and transcription processes happened simultaneously to preserve the contextual meaning and nuances of the participants’ responses. To ensure accuracy and credibility, a selected subset of the transcripts was reviewed by the team to cross-check for consistency and semantic equivalence with the original audio recordings. Any discrepancies were discussed and resolved through consensus. Audio recordings and transcripts were anonymized using unique identifiers ensuring that names and other potentially identifying details were removed. Consent forms were stored separately from study data. Transcripts and audio recordings were stored on password-protected drives with access restricted to the research team.

Data were analyzed using a deductive thematic analysis (31, 32), by three experienced qualitative researchers (PKM, AM and AK). A primarily latent content analysis approach was adapted (33) to ensure analysis were beyond surface-level descriptions and to interpret the underlying meanings, perceptions, and experiences related to factors influencing fistula care and surgery in Uganda. This approach was appropriate given the study's aim to explore provider perspectives and system-level dynamics.

The research team independently reviewed the transcripts to familiarize themselves in the data and identify key patterns, noting anticipated (deductive) themes that informed the development of an initial codebook. Identified codes were grouped into sub-themes and then organized into higher order themes including facilitators, barriers, adaptive mechanisms and recommendations for improvement of fistula surgery and care.

The coding framework was iteratively refined through constant comparison across the data, ensuring that deductively and generated themes were adequately captured. The final codebook was entered into Nvivo (version 14), which was used to code and manage the data. Transcripts were uploaded and coded using Nvivo 14 by dragging and dropping the relevant text segments into codes (themes). Themes were informed by the WHO system building blocks framework (34) and predefined categories of adaptation and recommendations.

The team met regularly throughout the process to review memo notes, iterate on the thematic map and agree on any emerging and re-emerging themes. Data saturation was assessed iteratively throughout the data collection process. Data collection stopped when no significantly new themes emerged from the interviews, and additional interviews provided limited new insights, indicating thematic saturation. Illustrative excerpts from the data were used to provide context while preserving anonymity; quotations are attributed only by role and site**.**

### Rigor and trustworthiness

This study employed four established criteria (credibility, transferability, dependability, and confirmability) to ensure rigor and trustworthiness (35, 36). Credibility was enhanced through site familiarization prior to data collection, iterative team discussions, and verification of interpretations against interview data. For transferability, an attempt was made to describe the characteristics of the participants and use maximum variability in sampling. To address the dependability, study processes were described to the team in detail by the first author (PKM). Discussions in research team meetings raised additional issues that were considered for confirmability. To aid potential transferability and replicability, we have adequately described our research objective, research design, data collection methods, analysis as well as provided information on the study setting and the participants. To ensure dependability and confirmability, the entire thematic analysis process was thoroughly reviewed by all three authors (PKM, AM, and AKM) and quotes were used to support themes. To facilitate transferability, a detailed description of the studied context and perceptions of the participants involved is included. All participants provided written informed consent prior to taking part in the interviews.

### Research team and reflexivity

The research team was interdisciplinary, consisting of midwives, nurses, social scientists, doctors and statisticians, which supported a broad understanding of the study context. The first author (PKM) brought a unique positionality, having worked in fistula programming for some time and holding a deep passion for improving fistula care services, which directly informed the study's aim to understand fistula surgery and care in Busoga region. To prevent PKM's knowledge and passion from influencing data collection, analysis and interpretation and results, the research team including research assistants underwent training that focused on understanding the study in detail, data collection and how team can reduce bias based on their different backgrounds and get the best of the study to inform policy for improved fistula treatment and care. This commitment to rigor was maintained through daily research team debriefing and feedback meetings during data collection and analysis, where the first author (PKM) also kept detailed reflexive notes. This collective commitment to reflexivity and the inclusion of diverse professional perspectives enabled effective collaboration, critical evaluation of each other's viewpoints and ultimately strengthened the overall rigor and objectivity of the study findings.

### Ethical considerations

The study received ethical clearance from the Biomedical and Research Ethics Committee (BMREC) and the Higher Degrees Research and Ethics Committee of the University of Western Cape (BMREC Reference Number: BM22/10/27), Uganda Christian University Research Ethics Committee (REF NO: UCUREC-2023–458), and the Uganda National Council for Science and Technology. We also obtained administrative clearance from the Ministry of Health and participating hospitals. All participants provided written informed consent and consent to audio record before participating in the study.

## Results

### Demographic characteristics of participants

Table 3 presents the demographic characteristics of the participants. For the IDIs, there were twelve (12) healthcare professionals that provide fistula services, aged between 28 and 66 years, participated, including eight females and four males. The majority were nurses (*n* = 4). Additionally, half of the participants (*n* = 6) had provided fistula care for more than five years. For the KIIs, eight (8) national-level stakeholders participated, including three males and five females, aged between 32 and 59 years.

**Table 3:** Demographic characteristics of participants.

Variable	Description	IDI	KIIs
Gender	F	8	3
	M	4	5
Age	25–34	5	1
	35–44	3	3
	45–54	2	2
	55 and above	2	2
	Secondary Education	-	1
Level of Education	Certificate	2	-
	Diploma	4	-
	Degree	2	-
	Master's degree	4	6
	Professor (University)	-	1
Cadre/Role in fistula care	Midwife	2	-
	Nurse	4	-
	Specialist doctor (surgeon/obstetrician/Urologist)	2	4
	Hospital administrator	2	-
	Hospital Director	2	-
	Implementor/Donor/NGO staff	-	2
	Policy (Ministry of Health)	-	2
Years in general care provision	1–5	4	1
	6–10	4	3
	11–19	1	3
	20 and above	3	1
Years in fistula care	1–5	6	2
	6–10	4	3
	11–19	1	2
	20 and above	1	1
Stakeholder category	National Expert	-	4
	Ministry of Health	-	2
	Partners/Donors	-	1
	Implementors/ NGO staff	-	1

### Thematic results

Table 4 presents the results according to six (6) main themes: 1) human resources, 2) equipment and supplies, 3) infrastructure, 4) financial resources, 5) access to services, 6) adaptation mechanisms. Within each theme, we report the key facilitators and barriers identified by participants, along with the adaptations employed and recommendations for strengthening fistula care at service delivery points. These themes were developed *a priori* based on the review of the literature. These mirror the critical systemic issues that affect the capacity to provide fistula surgery and care. We discuss the key facilitators and barriers to the provision of obstetric fistula surgery and care in each of the above themes. Additionally, we present the perceived adaptations, mechanisms for fistula care at the service delivery points.

**Table 4:** Shows the key themes and subthemes.

Theme	Facilitators	Barriers
Human resource	• Availability of trained fistula experts during camps • Training initiatives for experts, including urogynecology fellowship programs	• Inadequate staffing norms, leading to staff exhaustion from multitasking and long working hours • Few facility-based fistula specialists—facilities rely on external camp-based surgeons • High staff turnover due to transfers and the search for better opportunities • Low and delayed allowances leading to demotivation
Equipment and supplies	• Donor and partner support ensured the availability of consumables during camps • Leftover supplies used for routine services • Ministry of Health general grants and hospital absorption of utility costs	• Inconsistent and delayed supply of consumables outside camps • Lack of fistula-specific operating tables • Non-functional or inadequate ward equipment (e.g., adjustable beds) • Shortages of supplies, drugs and tests, forcing patients to buy/pay out-of-pocket
Infrastructure	• Modern theatres with stable power supply and backup generators • Presence of a dedicated fistula ward (in some facilities) • Clean and well-maintained sanitation facilities during camps	• Inadequate ward space leading to overcrowding, patients sleeping on floors, corridors or verandas, and premature discharges • Non-functional obstetric theatre facilities, like taps
Financial resources	• Donor funding, which supports camp operations • Host hospitals waived user fees and absorbed operational costs • Partners supported transport refunds, feeding and reintegration programs	• Heavy dependence on donor funding • Stagnant government funding for fistula care • Financial mismanagement, affecting mobilization, planning and services • Low remuneration for fistula staff affects morale
Access to services	• Community mobilization through radio, TV, outreach and other hospital platforms • Partnerships with NGOs, community health workers, and local networks for sensitization • Provision of transport and feeding • Survivor ambassadors and reintegration programs	• Cultural misconceptions linking fistula to witchcraft • Stigma and discrimination leading to mental health issues, and the fear of disclosure • Lack of awareness about fistula and services • Transport and subsistence costs—leading to out-of-pocket payments • Limited social support due to abandonment
Adaptation mechanisms	• Team-based task sharing and flexible scheduling—staff working extended hours to maintain service continuity in other routine services • Rapid role substitution during camps • Use of tents and repurposed spaces to manage bed shortages • Integration of counselling, transport refunds and social support • Continuous on-the-job training of multidisciplinary teams • Passion and intrinsic motivation to serve vulnerable women	

### Human resources

Participants reported that both hospitals had trained experts to conduct surgeries and provide fistula care, such as gynecologists, obstetric surgeons, anesthetists, nurses and midwives during the periodic camps. These experts perform the repairs and provide post-operative care during the camps. However, there were reportedly few stationed experts at the hospitals.

“What I can say is that even if we have few health workers, they have expertise in what they are doing. They are also ready to come in case of an emergency. For the doctors we have, the moment you call the doctor for support, they will always be there to review. Such things encourage us to continue serving the country” (HCW 05)

Leveraging the available healthcare workforce at the hospitals, who had been trained to conduct surgeries and provide post-surgery care, was highlighted as key to the sustainability of fistula care. It also ensured women with fistula received continuous post-operative care until full recovery.

“What has changed greatly are the staffing levels; with the restructuring the Government is doing, we shall have more surgeons and gynecologists on the ground.” (HCW 10)

Participants reported that there were initiatives to train and build the capacity of fistula experts, like the urogynecology fellowship at Mbarara University of Science and Technology. Unlike before when experts were trained on the job, these initiatives are vital in developing specialized skills and confidence of experts to provide fistula care, and increasing the number of trained surgeons and fistula nurses.

“Then, human resources are through our training and supported by donors. We train nurses, midwives, and theatre assistants in anesthesia through donations, and we are now training urogynaecologists in a fellowship program in Mbarara to train fistula surgeons.” (KII 08)

On the other hand, many participants highlighted that hospital staffing norms were inadequate, which affected continuous obstetric fistula surgery and care after the camps. Participants reported that there were few trained facility-based experts in fistula surgery and care.

“We have not been enough because you try and they go, and you find out that we need to try another group, so the two of us exhaust a lot because we have to be at the site at all times.” (HCW 06**)**

These few experts were forced to manage multiple roles during camps, as noted by one of the participants:

“The ones I know as of now are six, but again, if we were six and direct on fistula care, we could have done a great job, but we are not directly on fistula that much.” (HCW 05)

Due to a lack of dedicated in-house surgeons at the facilities, participants reported that hospitals relied on external camp-based experts for fistula surgeries. Even during fistula camps, experts were scheduled to be present only on specific days. Once they left, all pending cases were postponed until the next camp. This reliance on external specialists limited the continuity and accessibility of fistula care beyond the camps.

“Also, as I told you earlier, the lack of a Fistula surgeon is a barrier. We could be doing those routine surgeries, but we have to wait for camps because we lack the Fistula surgeries.” (HCW 05)

“Because even if you organize a camp here, do you have enough human resources to handle it? Sometimes, it means you require more people from out there to support you in the camp.” (HCW 09)

High staff turnover among trained fistula surgeons, nurses and midwives hindered effective and sustainable fistula care. Participants noted that trained fistula healthcare workers often left for better opportunities or were transferred to other facilities or different departments, leaving hospitals depleted of skilled specialists.

“And when you train them, they keep, even when you go to a hospital, the turnover is high. They keep moving from one ward to another. So, we still do not have enough human resources for fistula surgery.” (KII 01)

“They would have gotten green pastures somewhere, so we were left by the 2 of us, XX and I work 24/7…” (HCW 06)

Participants expressed dissatisfaction with allowances and remuneration for fistula care, which negatively impacted their morale and commitment. They noted that inadequate compensation led to frustration and demotivation among fistula healthcare workers.

“The money that they also give to us as nurses is not that good. It was actually too little, and we then complained, so they started sending us the money. Then they write email letting us know that sent such an amount of money, but still when it arrives, they will share it for all of us, and yet it is sent specifically for the nurses who have worked in the theatre” (HCW 06**)**

### Equipment and supplies

Most participants reported having essential equipment and supplies for fistula care, especially during fistula camps, including surgical supplies like catheters, needles, sutures, and antiseptics, beds and oxygen, etc. Support from partners, such as UNFPA, Engender Health, and the Uganda Childbirth Injury Fund, was highlighted as a critical factor in ensuring the availability of these resources during fistula camps.

“All I know is that our hospital has been equipped with most of the things, starting from the theatre; we have the things that we use. We have enough sets in the facility to help with the surgeries. For example, there is somewhere to sterilize the gowns from: all theatre requirements are available. In the wards, we have enough beds where our patients stay, even during those two weeks when patients must stay; we also have oxygen in the hospital just in case it's required.” (HCW 01)

Participants noted that leftover equipment and supplies from these camps were often repurposed for routine procedures and care, thereby enhancing the facility’s capacity to provide continuous services.

“Usually, the equipment lasts longer, and some to sundries are left over after the camp. So we use them for routine surgeries, and I believe those will enable us to start again” (HCW 10)

The Ministry of Health was perceived to play a crucial role in fistula care as it offered general grants to hospitals part of which is used for medical supplies. Hosting hospitals also provide infrastructure, waive service fees for patients, absorb operational costs for utilities (electricity, water, and sanitation) and provide logistical support, such as theatre supplies. This support, although indirect, was perceived to help in sustaining fistula services during camps.

“When these (fistula) patients are here, they use a lot of our resources, for example, electricity, water, and sanitation facilities, but we don't charge them. This is a paying hospital, but they don't pay any coins, so that's how we partner.” (HCW 02)

However, some participants, especially at Hospital A, highlighted the lack of or non-functional essential surgical and ward equipment, such as kidney dishes, galley pots and sterilization drums, as a significant barrier to fistula surgery and care.

“When we are going to give treatment, we need kidney dishes, galley pots and special drums but there are few.” (HCW 08)

Whereas critical consumables and medications were always provided during the fistula camps, participants reported delayed and inconsistent supply of these items, especially at the start and towards the end of the camps. The most mentioned items were sundries and oxygen. This forced health workers to improvise or request patients to purchase their own supplies.

“We have some rare drugs which are not always available, and they need to put in their money, and other patients need expensive investigations……we also tell them to use their own money.” (HCW 05)

Participants further noted that post-surgical care was compromised due to a lack of appropriate ward equipment like the lack of accessible hospital beds for fistula women.

“The beds we have now are not favorable to a mother having a VVF fistula. Imagine this mother climbing from bed to down and the energy she is going to use to come down.” (HCW 05)

### Infrastructure

Participants highlighted the presence of modern and well-equipped theatres at the hospitals as a key infrastructure facilitator in ensuring the success of fistula camps. These theatres had the necessary supplies and a stable power supply and standby generators to guarantee uninterrupted fistula surgeries and care.

“We have a nice theatre, every surgeon or donor that comes here says “This is a nice theatre”, and they always bring us camps, our nursing team, whichever surgeon comes and sees the nursing care sees that all patients that he/she has worked upon are going home well, they continue to come back here.” (HCW 03)

Participants at Hospital B reported that the presence of a dedicated fistula ward was crucial in providing effective pre- and post-operative treatment to women. This was perceived to enhance privacy, minimize stigma and discrimination for fistula women while on the ward.

“We have an established fistula ward, so whenever they want, they will find it, and whenever they come to schedule, it will be there to accommodate the patients. May be the kits will accommodate the visiting surgeons. Basically, we are ready for the water that patients can use, so those are the ones I can think about.” (HCW 01)

Participants reported that the hospitals had adequate well-maintained toilets during fistula camps, which were kept clean and in good condition to ensure suitability for use by the mothers.

“By the way, our toilets are very clean, and with that, I appreciate the cleaners; they usually keep them very clean, and they check them throughout the day to see that they are very clean.” (HCW 08)

The infrastructure barriers included the lack of adequate ward space at both hospitals during fistula repair camps. Participants reported that this led to overcrowding, even at Hospital A, which did not have a dedicated fistula ward. Consequently, many women were forced to sleep on ward floors, verandas or hallways especially before surgery. Healthcare workers were also forced to discharge patients prematurely, before full recovery to create space for incoming patients.

“As of now, the ward space is not enough. Right now, you see a few people, but we always have many people..” (HCW 05)

“But now they all sleep inside because there are small rooms in there where we can squeeze them, the remaining ones then sleep in the corridors… now the tent has worn out that we used to give them.” (HCW 06)

Some participants reported that facilities in the theatres designated for obstetric and gynecological surgeries, including fistula repairs, were either defective or overwhelmed with other surgical cases.

“We indeed have an obstetric theatre, but it's not functioning and not up to standard. Currently, it's non-functional because it's not conducive, congesting the main theatre.” (HCW 12)

### Financial resources

Participants reported that fistula care and surgeries heavily relied on the financial support provided by donors and organizations, including Fistula Foundation, UNFPA, Health Girona Foundation, Medicine for Humanity and the Government of Iceland. They noted that these organizations largely finance surgeries including ensuring the availability of equipment and supplies, facilitation of experts and other care workers during camps and training programs for medical personnel to build the capacity in providing care.

“First, we usually get finances from donors. UNFPA, through the Ministry of Health, and other NGOs, like Health Girona Foundation, Fistula Foundation, and Medicine for Humanity. So, fistula surgery is mainly financed through donors. For equipment, still through donor donations.” (KII 08)

Participants reported that some partners, like Uganda Village Project and Terrewode, among others, facilitated patients while on the ward and during post-operative care. They offered transport refunds, food and reintegration programs for patients further easing the treatment and recovery process.

“Food, transport refund, that's all from the Uganda village project then the integration program we keep them for the 2 months so that they get the services they are supposed to get as discussed before.” (KII 05)

Financial barriers reported by participants included hospitals heavily depending on donor support for financial resources while Government health budgets remained stagnant. They reported insufficient Government funding particularly for obstetric fistula care.

“Our budgets have not been increasing, and government funding has been stagnant for a while, so financing this service is a challenge; that is why, most times, it is partner-supported, which is the other barrier we have.” (HCW 04)

Participants also highlighted financial mismanagement which hindered patient mobilization efforts and generally affected quality of fistula care. Poor fund allocation caused sudden plan changes. Consequently, this reduced the effectiveness of these activities.

“Money has brought problems here because 1) it's the mobilization that goes first whereby the UCBIF sends the money to use for mobilization, so when this money reaches the office, getting it is hard.” (HCW 06)

### Accessibility

Accessibility facilitators included ongoing community engagement and promotion of fistula services particularly when camps are scheduled at both hospitals. This is carried out through radio and TV programs, as well as outreach activities, which are essential for raising awareness and mobilizing patients regarding the fistula camps. As a result, women living with fistula were informed about the camps and the services available to them.

“It's mainly on the radio and these outreaches. For example, when we are going to have a camp, we move from facility to facility, and the midwives help us teach during antenatal clinics which also enables them to get that information.” (KII 05)

Participants reported that fistula camps offered a unique healthcare seeking experience for women with fistula one that is not typically the case during routine care at public hospitals. These camps enabled facilities to plan and ensure the availability of adequate supplies, essential medicines and human resources needed for successful fistula care. In partnership with various partners like Uganda Village Project, participants reported that efforts were made to reach out to patients in need of fistula treatment. This was done through lower-level healthcare facilities and community healthcare workers as well as local networks like village leaders who mobilize mothers in need of fistula surgeries within the catchment area.

“We also have health workers we connect with from UVP (Uganda Village Project) who look for them from various areas, they get them from Busia, Tororo, Iganga, Jinja, more so in the Busoga region.” (HCW 06)

During fistula camps, hospitals used those networks and sometimes took advantage of channels like WhatsApp groups to spread information about upcoming camps. Participants reported that these had been effective in sensitizing women and informing them about fistula camps or services.

“Yes, you can give a phone call, we have a WhatsApp group, you post there… The WhatsApp group is for women and childcare, so any issue concerning a woman or a child, you post there, and they can.” (HCW 08)

The provision of transportation and feeding during care was perceived as an enabler to access fistula care. Participants reported that most women suffering from fistula were economically disadvantaged, and providing them with transportation to facilities and feeding eased their care-seeking experiences.

“We give them transport refunds and transport is used to bring them. Sometimes, we go to pick them up from a health facility where they gather and we transport them in a vehicle that brings them to this facility. After surgery, we give them a transport refund back home.” (HCW 03)

Participants reported that social integration initiatives by hospitals and partners not only support survivors in regaining their confidence and returning to their normal social life but also build confidence in others living with such conditions to seek care.

“Social reintegration, we have also improved because previously, we never had that component. When the mothers are repaired, we go ahead and follow them up in the community to ensure that they are reintegrated back into society.” (KII 03)

Through these initiatives, fistula survivors were empowered and worked as ambassadors to mobilize and motivate others to seek surgeries by sharing successful and inspiring stories especially about the fistula camps and services offered.

“Now for the UVP that we collaborate with, so for them, they mobilize them, they have a fistula ambassador and take them to Namugari center, where they collect all patients, and that's where they screen them before they come here.” (HCW 06)

However, participants also reported significant access barriers to fistula surgery and care for women living with the illness. Cultural misconceptions, stigma, and discrimination significantly hindered women's ability to seek fistula treatment. Participants noted that beliefs associating fistula with witchcraft or supernatural forces negatively affected women's perceptions and experiences with fistula, hence discouraging many women from seeking fistula surgery.

“Most women still think that when you have a fistula, it means that they bewitched you. I believe that if they come out and join the fistula groups and tell them about this, you can clear their minds from that.” (HCW 05)

Participants also noted that while surgeries were free, the expenses associated with transportation and hospitalization present major obstacles for women with fistula, hence they are unable to access camps.

“No, surgeries are free of charge, but where the problem comes from is when most people do not have money to transport them from the villages to the hospital, no money to cater for their feedings during the period they will be in the hospital” (HCW 08)

Although camp services were provided at no cost for women with fistula, participants reported that the hospitals often lacked essential medications and medical tests, particularly at Hospital A. Women often had to pay out-of-pocket for expensive medical tests and scans impacting their care experiences and discouraging others from seeking fistula surgery.

“We have some rare drugs which are not always available, and they need to put in their money, and other patients need expensive investigations and difficult scans that they have to do (out of pocket).” (HCW 05)

Participants indicated that patients were reluctant to disclose their conditions due to fear of stigma which also affected their ability to seek care. One respondent highlighted.

“If you are with a client and that client knows that a certain client has known their information, she can feel small… No one would want to know their condition.” (HCW 08)

There were also issues related to the lack of support networks for women living with fistula, which affected their access to treatment and care. Discrimination, stigma and social isolation led to breakdowns in relationships with many women experiencing abandonment by their partners, families and friends. This impaired their mental health and complicated their access to medical attention.

“Most of these clients have taken so long with this problem, and they give reasons for not coming; financial or social problems and at times, most of them, once they get those problems, their husbands leave them.” (HCW 08)

Additionally, there were also mental health challenges associated with fistula. Participants reported that the psychological effects of living with obstetric fistula led to feelings of shame and contributed to social isolation as emphasized by one of the key informants, all of which affected their ability to seek formal care.

“Every fistula patient has a mental illness… Let me use you as an example; right now, you're fine and normal, but imagine getting such a disease, and the urine starts to leak out of nowhere. Right now, you can get a deal and run for it, but if the urine starts leaking, will you go, or will you be free with people?” (HCW 05)

Participants highlighted a significant lack of awareness about obstetric fistula and the available care options among women living with fistula, creating a barrier to accessing treatment and services.

“Another challenge we have is sensitizing the community. For example, you might find out that we have a few fistula patients from Kamuli district, yet there are many in other districts. Our role as fistula workers or midwives is to go to those places and sensitize them more about fistula because most of them need our services to repair them.” (HCW 05)

### Adaptation mechanisms

Participants reported that healthcare workers in fistula care embraced a collaborative approach to ensure uninterrupted service delivery in response to human resources shortages. This teamwork enhanced the quality and impact of fistula care for women.

“We work as a team; we would not have challenges where you need help and cannot find help. The teamwork is amazing; we always have skilled people, so you cannot get stuck. So, I think what has been helping me is teamwork. Teamwork between the specialists working, the nurses and the anesthetist, the nurses doing pre-and post-operative care, during the surgeries, so the teamwork has been helpful.” (KII 7)

They adjusted their schedules, shared responsibilities, and stepped into multiple roles of routine care and the camp services when needed.

“Yes, although you can do both at the same time during the day or work day here and work night there. However, some people working here during the day work night shifts there, meaning that she is working for 24 h non-stop.” (HCW 06).

The fistula experts commit to working longer hours to extend fistula services and treatment to fistula clients, especially during camps. This ensured the success of fistula camps and efficient services to patients. One of the health workers highlighted:

“For all these years, there hasn't been any excuse from the workers, so what I do during the days of my hospital duties, I make sure I wake up early to cover it up; we first finish rounds very fast, then I join the Fistula team.” (HCW 02)

Participants reported passion and a desire to save the most vulnerable women as major motivators for healthcare workers to provide fistula care. The dedication of fistula experts fostered empathy ensuring women received the best surgeries and care, and felt valued and cared for throughout the treatment process. Emotional rewards from witnessing recovery and reintegration, restoring patients’ dignity, self-esteem, hope and fuel their commitment to health workers.

“As I said, it's a passion. So, the first enabler is passion and liking what to do because fistula surgery is not like any other surgery, like caesarean section, where you do surgery to get money. Here, there is no money attached to it. You do it out of passion and out of helping the patient because there is nothing in return, you're going to get in terms of monetary value.” (KII 08)

To address patients’ psychological challenges, participants reported that they extended services beyond clinical care. Counselling sessions, transport reimbursements, and community engagement were strategies used to bridge socioeconomic gaps. A healthcare worker noted:

“What we always do here is, for example, now, in relation to a transport refund for a patient. If a patient has failed to get transport to bring them, we always encourage them so that they can get transport to the facility… just get transport to get you to the facility, and we shall give you a refund to take you back, and the feeding is at the hospital”. (HCW 07)

Participants noted that hospitals adapted remarkably to limited bed capacity by creatively repurposing existing spaces and installing temporary structures like tents to accommodate all patients.

“For infrastructure, we used tents where we had overwhelming numbers; for example, we put the tent out, and for the patients who are not yet booked but have VVF, so when they come, they stay in a tent that is well-made and good enough for you to wait for your day when they are screened and ready for surgery.” (HCW 12)

Participants also reported that effective human resource training was essential for addressing the challenges in fistula surgery. They emphasized that a welltrained workforce forms the foundation of a successful fistula treatment team.

“A surgeon can't be successful if you don't have a team. So, we do so much training of fistula teams, not only surgeons but trained nurses, both theatre nurses and ward nurses. We train anesthetists; we train counsellors. So that's one of the coping mechanisms of how we try to go about this human resource issue.” (KII O8)

Participants reported that social integration programs were effective in fighting stigma and discrimination surrounding fistula for affected women. Community sensitization and socioeconomic reintegration programs were perceived to empower them to reintegrate into the communities.

“When they (fistula patients) are done with treatment, we take them for reintegration for a period of 2 months and involve them in vocational activities like tailoring, bead making, basket weaving to restore hope into them… It depends; some communities will discriminate, so we go as a team to sensitize the specific community with support from the chairpersons.” (KII 05)

Concerning financial challenges, participants reported that the hospitals primarily relied on partnerships and Government support to secure funding of fistula camps and all fistula care services, including social integration activities. One national key informant highlighted:

“We always try to look for partners. We run to the government through the Minister of Health so that we get some funds to help us do these fistula surgeries. So, it is mainly through partners and training.” (KII 08)

“They (the external support team) have always carried many lamps and gargles; for example, this isn't mine, but I’ve been given to use for today, and they even come with things to support the people they have found at the facility.” (HCW 01)

## Discussion

This study explored service providers’ perceptions of facilitators, barriers, adaptive mechanisms and recommendations for improving care for the provision of obstetric fistula surgery and care in the Busoga Sub-Region, Uganda. The discussion is structured around facilitators, barriers, and adaptive mechanisms across the themes of human resources, equipment and supplies, infrastructure, financial resources, access to services, adaptation mechanisms and recommendations for improving obstetric fistula care.

The findings highlight a healthcare system that has developed an adaptive model of fistula repair camps to respond to the growing need of fistula treatment services. However, the system continues to face persistent structural constraints that limit the availability, continuity and sustainability of routine fistula services.

Among the key facilitators that enabled obstetric fistula care in Uganda was the stewardship of the Ministry of Health. This underscores the critical role of government commitment in translating policy recommendations into sustainable service delivery. Continued coordination and financial support by the government are therefore essential for expanding fistula surgery and care and ensuring the long-term sustainability of fistula repair services across all regions. Uganda was one of the first countries to develop the National Obstetric Fistula Strategies (13, 14). The strategies aimed at fistula prevention, treatment, re-integration and monitoring performance of fistula programs across the country, and indicator that fistula was recognized as a public health problem. Many countries have developed national strategies and the presence of comprehensive policy frameworks in affected countries is a commitment to ensure service availability and a critical foundation for action (8, 11).

Another key facilitator was the presence of trained, dedicated and resilient teams. Providers emphasized the motivation, commitment and sense of moral responsibility among the trained surgeons, nurses, midwives and other theatre staff who continued to offer services amidst inadequate resources. While this intrinsic resilience supports service delivery, it also highlights the need for professional recognition and deliberate efforts to address the infrastructural and resource deficits that currently rely on their personal sacrifice to the delivery of fistula services. This intrinsic resilience mirrors findings from studies on specialist surgical services in constrained systems where individual champions often sustain critical services in the absence of strong systemic support (37–41).

The FIGO Fistula Surgery Training Initiative underscores that training facility-based team, including fistula surgeons, nurses and midwives, is critical for routine fistula services and that clinical champions are central to strengthening the fistula treatment pathway (22, 23). Similarly, Pollaczek and colleagues demonstrated that building fistula treatment networks in Kenya facilitated collaboration across hospital and community actors including fistula surgeons and community workers to enhance long-term outcomes for women living with fistula (42, 43).

Participants emphasized that having a sufficient number of trained local staff was critical for facilitating successful fistula repairs. This suggests that integrating fistula care and training into the services provided by local hospitals is essential for sustainability (4, 23, 27). Dedicated multidisciplinary teams are required to provide pre-operative, intra-operative and post-operative care to achieve quality outcomes. However, reliance on personal commitment makes these services fragile and vulnerable to staff turnover and burnout. Additionally, shortages of trained fistula surgeons and nurses, high turnover rates, and inconsistent engagement of trained staff emerged as significant concerns. These findings align with previous studies that show training alone is insufficient without strategies for staff retention, protected time for fistula work, and supportive working conditions (44–46).

The provision of “free” fistula surgical repair services, largely financed through donor support, was also a key facilitator that reduced the financial burden for women seeking fistula care (18, 38, 47). The free fistula repair supported by UNFPA, NGOs and the Ministry of Health significantly improve and restores the dignity of women affected by fistula (25, 48, 49). However, the findings highlight that financial access for patients does not automatically translate into system readiness to deliver care or ease service access. Hospitals relied heavily on donor funding with little or no budget allocations from the government for fistula care (15). While donor support enables life-saving surgeries, overreliance on external funding undermines sustainability and long-term planning. Similar patterns have been observed in other surgical domains, where vertical funding supports high-volume outreach activities but leaves routine services under-resourced and unprepared for continuous care (50).

Infrastructure and equipment gaps further constrain service delivery. Hospitals lacked dedicated theatre space, fistula-specific operating tables, adjustable beds, and consistent access to essential consumables. According to the WHO guiding principles for fistula management (8, 9), successful repair requires a functioning system rather than episodic availability of surgical inputs. The absence of these components restricts hospitals’ ability to offer continuous fistula care even when skilled personnel are present.

A central, yet paradoxical finding of this study is the dual role of fistula repair camps. On one hand, providers viewed camps as a necessary and pragmatic adaptation to systemic challenges. Camps enable the rapid mobilization of resources, concentrated surgical output, mentorship opportunities, patient mobilization, and visibility for fistula programs. Similar benefits have been documented in eye surgery camps, cleft lip and palate missions and reconstructive surgery outreach (47, 51–53). The arrangements and coordination models for obstetric fistula camps are functionally similar to those for eye surgery, cleft lip and palate and reconstructive surgery.

On the other hand, the over reliance on camps undermines the sustainability of routine fistula services and directly affects the reduction of the large case backlog in the country. Camps were described as episodic, donor-driven, and poorly integrated into hospital systems. Other countries, such as Ethiopia, have put in place strategies to ensure fistula services are integrated into the routine repairs (54). This tension reflects broader debates in global surgery that, while camps can reduce backlogs and reach underserved populations, they may disincentivize government investments in routine services and weaken continuity of care. The findings suggest that camps function less as a preferred service delivery model and more as a symptom of underlying system failure. Therefore, the effectiveness of camps should not be measured by the surgical volumes and successes but by how well they contribute to the broader health system strengthening efforts (47).

A programmatic shift towards permanent and fully integrated fistula services in selected hospitals is needed to ensure continuous and holistic care. Health systems literature consistently emphasizes that sustainable surgical care requires integrated approaches across governance, financing, workforce, infrastructure and service delivery (55). Experiences from countries that have transitioned from camp-based models to routine service provision such as Ethiopia's integration of fistula repair into selected regional hospitals demonstrate the importance of long-term investment in institutional capacity (55). Camps could then be repositioned as complementary, supporting training, mentorship, backlog reduction, and outreach, rather than serving as the primary mode of service delivery (47).

The findings of this study reinforce the urgent need for targeted interventions to improve the readiness and capacity of health facilities in the country to provide routine obstetric fistula surgery and care. Addressing the identified barriers, particularly human resource shortages, infrastructural gaps and supply chain issues, is paramount in restoring dignity for the women with fistula. Strengthening systems capacity to repair and care for clients with obstetric fistula and integrating treatment and care into routine service delivery are crucial steps towards achieving the vision of ending fistula by 2030.

### Strengths of the study

The use of in-depth interviews in this qualitative study enabled a nuanced and in-depth exploration of facilitators, barriers, adaptations mechanisms and recommendations to improve fistula care and treatment from the perspectives of service providers. The inclusion of a multidisciplinary group of participants including healthcare providers involved in fistula care such as fistula midwives, nurses, specialist doctors such as surgeons, obstetricians, and urologists, hospital administrators, hospital directors, implementers, donors, partners, and policymakers from the Ministry of Health ensured the validity of the interviews conducted and the results generated, improving the credibility of the findings.

### Limitations of the study

The study had limitations that should be considered when interpreting the findings. First, as a qualitative study conducted in two facilities within the Busoga sub-region, the findings may not be statistically generalizable and limits the transferability of the findings to other fistula care and treatment settings in Uganda or other contexts. Regardless, the great insights generated by this study analytically transferable to similar healthcare system contexts. Since the interviews were conducted within general hospital settings, there was the possibility of social desirability bias, as participants could have moderated their responses in order not to seem to implicate themselves or their colleagues. Although the research team made efforts to ensure privacy and confidentiality, this may have led to underreporting or negative experiences or systemic challenges. The study involved service providers involved in fistula camps, which may have shaped findings towards camp-based perspectives and underrepresenting experiences related to routine service provision of fistula care. Also, the absence of patient perspectives means findings reflect a one-sided view, from only provider, and future studies should triangulate with patient experiences to provide a more comprehensive understanding of fistula care. Besides that, the timing of the study during repair camps may have introduced systematic biases. Participants’ perceptions of resource availability, staffing, and system functioning during fistula camps could differ significantly from their experiences with routine (non-camp) services. This variation may have influenced the findings of the study. Finally, although rigorous translation was employed, subtle nuances in meaning may have been lost due to lack of back-translation, which could influence the interpretation of some findings. The team acknowledges this as a limitation, however the team were experienced bilingual researchers. The team shared among themselves a selected subset of the transcripts to cross-check for consistency and semantic equivalence with the original audio recording.

### Implications for practice

The findings suggest that a shift toward hybrid models may be warranted and merits further investigation. The camps mode can be used as an opportunity for training and mentorship of fistula surgeons and teams and clearance of backlog. The provision of routine fistula care and treatment can be improved at regional, district level hospitals and health center levels, making fistula surgical teams an integral part of a hospital's service and through addressing other detailed barriers documented in this study but also ride on the identified facilitators for delivery of services.

The findings also suggest policy shifts in redefining access for fistula care and surgery not only based on financial waiver “free surgery” to ensuring system readiness including availability of fistula teams, supplies, equipment, infrastructure and accountability at all levels. It is critical to introduce staffing norms and compensation packages specifically for facility-based fistula teams as the current reliance on passion and moral motivation is non-sustainable. In addition, establishing dedicated domestic resources for fistula specific essential supplies, equipment and associated infrastructure is critical in order to end the structural trap of donor dependency.

## Conclusions

This study highlights the key facilitators, barriers and adaptation mechanisms required to improve the provision of obstetric fistula care in the subregion. While the short term, life-changing access provided by fistula camps remains a valuable stop gap measure, their continued necessity ultimately reflects underlying, unaddressed deficiencies within the routine health system. Achieving equitable and sustainable fistula care requires a strategic shift, moving beyond temporary arrangements to the sustained strengthening of routine services. This requires a comprehensive, system-level approach that directly addresses systemic structural, financial, and sociocultural barriers. Policy efforts must therefore prioritize dedicated, long term investment in core health system components, underscoring the need for improved infrastructure, reliable equipment and supplies, and continuous training for specialized fistula surgical teams.

### What is already known on this topic

- Barriers to accessing treatment and facility care from the perspective of women living with obstetric fistula in low-income countries are well reported.

### What this study adds

- This study addresses a significant gap by documenting the *facilitators, barriers and adaptations related to the provision of obstetric fistula surgery and care* from service provider perspectives in Uganda.
- These findings highlight the paradoxical role of fistula camps as temporary adaptive mechanisms that function as substitutes for routine obstetric fistula repair, reinforcing a model that treats symptoms rather than strengthening underlying health system structures.
- The study contributes a seldom-described perspective to the literature on health system resilience, showing how service providers compensate for critical structural weaknesses within fragile health system. These adaptions include passion and interest-driven service provision for, role substitution, moral motivation, and even the use of improvised facilities like tents

### How this study might affect policy shifts and practice

- There is urgent need to transition from episodic camps to a more integrated and sustainable model through capacity building for routine fistula repairs.
- Fistula camps should reposition and strengthened as platforms for training, mentorship, and backlog clearance.
- It is critical to introduce staffing norms and appropriate compensation packages specifically for facility-based fistula teams, as current reliance on passion and moral motivation is non-sustainable.

## Data Availability

The raw data supporting the conclusions of this article will be made available by the authors, without undue reservation.

## Supplementary material

Supplementary Table S2 Consolidated criteria for reporting qualitative studies (COREQ): 32-item checklist
