# Supplementary material for: Perceptions of facilitators, barriers and adaptations for provision of obstetric fistula surgery and care: a qualitative descriptive study of service providers in selected hospitals in Busoga sub-region, eastern Uganda
Source: Front Glob Womens Health. 2026 Jun 3;7:1813512. doi: 10.3389/fgwh.2026.1813512 (PMC13272310; doi:10.3389/fgwh.2026.1813512)
Supplement: Supplementary Table S2 — Consolidated criteria for reporting qualitative studies (COREQ): 32-item checklist [file Table1.docx]

**Supplementary material**

**Indepth Interview Guide**

| B1. Sex of the respondent |
| --- |
| B2. Age of the respondent |
| B3. Highest level of education of the respondent |
| B4. Position of person interviewed? |
| B5. If B4==1, what is the doctor’s specialization? |
| B6. Are you a fistula surgeon? |
| What is your Position/Designation (SPECIFY) |
| B7. How many years of experience do you have in providing health services to patients? |
| B8. Tell me about your experience in treating fistula patients? (probe for number of years, number patients treated) |
| What are the successes in treating fistulas? |
| What challenges have you faced while treating fistulas? |
| B9. What are the **facilitators/enablers for provisi**on of fistula services? (Probe as much as possible but do not lead the respondent—human -Human Resource, Infrastructure (e.g. dedicated ward, theatre), Financial resources and social constraints etc.) |
| What are the Human Resource facilitators for provision of fistula surgery |
| What are the infrastructure facilitators for provision of fistula surgery |
| What are the equipment and supplies facilitators for provision of fistula surgery |
| What are the financial resources facilitators s for provision of fistula surgery |
| What are the access facilitators/enablers s for provision of fistula surgery from the perspective of the client |
| B10. What are the barriers for provision of fistula services  (Probe as much as possible but do not lead the respondent - Human Resource, Infrastructure, Financial resources, social constraints etc.) |
| What are the Human Resource constraints and barriers for provision of fistula surgery |
| What are the infrastructure constraints and barriers for provision of fistula surgery |
| What are the equipment and supplies constraints and barriers for provision of fistula surgery |
| What are the financial resources constraints and barriers for provision of fistula surgery |
| What are the access constraints and barriers for provision of fistula surgery from the perspective of the client |
| **Coping/Adaptation mechanisms** |
| B11. What are the coping mechanisms or adaptations that you have noticed or practiced in your career to enable you to overcome the Human resource barriers mentioned in B10 |
| What are the coping mechanisms or adaptations that you have noticed or practiced in your career to enable you to overcome the infrastructure barriers mentioned in B10 |
| What are the coping mechanisms or adaptations that you have noticed or practiced in your career to enable you to overcome the equipment and supplies barriers mentioned in B10 |
| What are the coping mechanisms or adaptations that you have noticed or practiced in your career to enable you to overcome the financial barriers mentioned in B10 |
| B12. What recommendations do you suggest that can help to improve access to and the quality of fistula treatment services in Uganda? (Probe as much as possible but do not lead the respondent - Which barrier / facilitating factor is the recommendation targeting, who should implement the recommendation? When is the recommendation supposed to be implemented?) |
| Recommendation for Human resource barriers |
| Recommendation for financial barriers |
| Recommendation for infrastructural barriers |
| Recommendation for equipment and supplies barriers |
| Recommendation for access barriers |
| Who should implement the recommendations; mention |
| When is the recommendation supposed to be implemented? |
| Comments and additional notes |
