# Supplementary material for: Perceptions of facilitators, barriers and adaptations for provision of obstetric fistula surgery and care: a qualitative descriptive study of service providers in selected hospitals in Busoga sub-region, eastern Uganda
Source: Front Glob Womens Health. 2026 Jun 3;7:1813512. doi: 10.3389/fgwh.2026.1813512 (PMC13272310; doi:10.3389/fgwh.2026.1813512)
Supplement: Supplementary file 2 [file Datasheet1.pdf]

Supplementary material

Indepth Interview Guide

B1. Sex of the respondent
B2. Age of the respondent
B3. Highest level of education of the respondent
B4. Position of person interviewed?
B5. If B4==1, what is the doctor's specialization?
B6. Are you a fistula surgeon?
What is your Position/Designation (SPECIFY)
B7. How many years of experience do you have in providing health services to patients?
B8. Tell me about your experience in treating fistula patients? (probe for number of years, number patients treated)
What are the successes in treating fistulas?
What challenges have you faced while treating fistulas?
B9. What are the **facilitators/enablers for provisi**on of fistula services? (Probe as much as possible but do not lead the respondent—human -Human Resource,

Infrastructure (e.g. dedicated ward, theatre), Financial resources and social constraints etc.)
What are the Human Resource facilitators for provision of fistula surgery
What are the infrastructure facilitators for provision of fistula surgery
What are the equipment and supplies facilitators for provision of fistula surgery
What are the financial resources facilitators s for provision of fistula surgery
What are the access facilitators/enablers s for provision of fistula surgery from the perspective of the client
B10. What are the barriers for provision of fistula services<br>(Probe as much as possible but do not lead the respondent - Human Resource, Infrastructure, Financial resources, social constraints etc.)
What are the Human Resource constraints and barriers for provision of fistula surgery
What are the infrastructure constraints and barriers for provision of fistula surgery
What are the equipment and supplies constraints and barriers for provision of fistula surgery
What are the financial resources constraints and barriers for provision of fistula surgery

What are the access constraints and barriers for provision of fistula surgery from the perspective of the client
**Coping/Adaptation mechanisms**
B11. What are the coping mechanisms or adaptations that you have noticed or practiced in your career to enable you to overcome the Human resource barriers mentioned in B10
What are the coping mechanisms or adaptations that you have noticed or practiced in your career to enable you to overcome the infrastructure barriers mentioned in B10
What are the coping mechanisms or adaptations that you have noticed or practiced in your career to enable you to overcome the equipment and supplies barriers mentioned in B10
What are the coping mechanisms or adaptations that you have noticed or practiced in your career to enable you to overcome the financial barriers mentioned in B10
B12. What recommendations do you suggest that can help to improve access to and the quality of fistula treatment services in Uganda? (Probe as much as possible but do not lead the respondent - Which barrier / facilitating factor is the recommendation targeting, who should implement the recommendation? When is the recommendation supposed to be implemented?)
Recommendation for Human resource barriers

Recommendation for financial barriers
Recommendation for infrastructural barriers
Recommendation for equipment and supplies barriers
Recommendation for access barriers
Who should implement the recommendations; mention
When is the recommendation supposed to be implemented?
Comments and additional notes
